# Pain Hypersensitivity in a Mouse Model of Marfan Syndrome

**DOI:** 10.3390/antiox15010080

**Published:** 2026-01-08

**Authors:** Rebecca Kordikowski, Joana Coutinho, Ignacio Martínez-Martel, Clara Penas, Beatriz Martín-Mur, Belén Pérez, Francesc Jiménez-Altayó, Olga Pol

**Affiliations:** 1 Grup de Neurofarmacologia Molecular, Institut de Recerca Sant Pau (IR SANT PAU), Sant Quintí 77-79, 08041 Barcelona, Spain; 2 Institut de Neurociències, Universitat Autònoma de Barcelona, 08193 Barcelona, Spain; 3 Department of Pharmacology, Therapeutics, and Toxicology, School of Medicine, Universitat Autònoma de Barcelona, 08193 Cerdanyola del Vallès, Spain; 4 Department of Cell Biology, Physiology and Immunology, Universitat Autònoma de Barcelona, 08193 Cerdanyola Del Vallès, Spain; 5 Centro de Investigación Biomédica en Red Sobre Enfermedades Neurodegenerativas (CIBERNED), Instituto de Salud Carlos III, 28029 Madrid, Spain; 6 Red Española de Terapias Avanzadas (RED-TERAV), Instituto de Salud Carlos III, 28029 Madrid, Spain; 7 Centro Nacional de Análisis Genómico (CNAG), Universitat de Barcelona, 08028 Barcelona, Spain; 8 Centro de Investigación Biomédica en Red de Enfermedades Cardiovasculares (CIBERCV), Instituto de Salud Carlos III, 28029 Madrid, Spain

**Keywords:** pain, Marfan syndrome, thoracic aorta aneurysm, neuroinflammation, oxidative stress, muscular deficits

## Abstract

Marfan syndrome (MFS) is a genetic disorder caused by mutations in the fibrillin-1 (Fbn1) gene, leading to structurally abnormal elastic fibers and diverse clinical manifestations. Aortic root dilation represents the most serious threat, often requiring prophylactic surgical repair. Emerging evidence suggests that MFS patients experience increased pain sensitivity, contributing to functional impairment and reduced quality of life. Here, we used C57BL/6 wild-type and Fbn1^C1041G/+^ (MFS) mice to examine brain transcriptomics, aortic histology, nociceptive behaviors, grip strength, and spinal cord gene expression in both sexes at 2, 4, 6, 8, and 16 months of age. Transcriptomic analysis revealed reduced activation of pain-related pathways in young males and aged females, with a reversal in aged males, suggesting age- and sex-dependent differences in pain modulation. Behavioral testing showed progressive mechanical and thermal hypersensitivity in MFS mice, with cold allodynia as the earliest manifestation with late-onset muscle weakness. In the spinal cord of 16-month-old MFS mice, increased expression of key excitatory and nociceptive markers was observed, consistent with the pain hypersensitivity phenotype. In addition, aged female MFS mice exhibited elevated spinal expression of pro-inflammatory cytokines, inducible nitric oxide synthase, and Nox4, whereas males showed increased transforming growth factor-β1 and Nox1, reflecting distinct inflammatory and oxidative stress profiles. These findings demonstrate that Fbn1^C1041G/+^ mice reproduce pain hypersensitivity and muscle deficits observed in MFS patients, supporting their use as a preclinical model. Our results suggest that enhanced spinal excitatory/nociceptive signaling, together with neuroinflammation and oxidative stress, contributes to sex- and age-specific pain mechanisms in MFS.

## 1. Introduction

Marfan syndrome (MFS, OMIM #154700) is a rare genetic disorder of connective tissue caused by mutations in the fibrillin-1 (*FBN1*) gene [1]. The FBN1 gene encodes fibrillin-1, a glycoprotein that plays a crucial role in the extracellular matrix (ECM). Fibrillin-1 forms microfibrils that serve as scaffolding for elastin fibers, ensuring the proper elastic recoil of connective tissue. When fibrillin-1 is dysfunctional or deficient, the assembly and stability of elastic fibers are impaired, leading to fragmented and structurally compromised fibers [1]. As a result, MFS manifests in a wide range of ocular, skin, skeletal, respiratory, and cardiovascular manifestations, with significant clinical variability [2]. Cardinal features include *ectopia lentis*, skeletal abnormalities such as scoliosis, disproportionately long extremities, and chest wall deformities. The most serious complication is dilation of the aortic root, which predisposes patients to dissection [1]. In fact, thoracic aortic aneurysm and dissection remain the leading causes of death in MFS [3].

Medical advances in the treatment of cardiovascular manifestations, such as the use of β-blockers and, most importantly, surgical repair of the aortic root, have significantly increased the life expectancy of individuals with MFS. However, the overall quality of life of these patients still requires improvement. Chronic pain has emerged as a clinically relevant concern in MFS, particularly as increased longevity extends the duration of exposure to painful skeletal manifestations [4]. Pain prevalence in individuals with MFS has been reported to vary substantially across studies and cohorts. While some investigations describe relatively high rates of pain (47–92%) [5], other work indicates a lower prevalence within certain MFS populations [6]. Together, these findings highlight the heterogeneity of pain experiences in MFS and underscore the need for further research to clarify the factors contributing to this variability. In patients with MFS, age is significantly correlated with increased pain and reduced physical function [7], underscoring the importance of early detection to prevent progression. Previous evidence suggests that chronic pain in MFS is associated with fatigue, educational and occupational challenges, sleep disorders, and psychological comorbidities such as depression and anxiety [8]. Addressing pain is crucial for reducing disease burden, yet few studies have described treatment options for individuals with MFS. In some cases, musculoskeletal deformities (e.g., chest deformities, scoliosis, dural ectasia) can cause or contribute to pain [8]. However, the overall etiology of pain in MFS remains complex, and there are no current approved therapies. Patients with MFS often seek treatment for diffuse musculoskeletal pain, which is often difficult to treat effectively, likely due to the absence of a specific triggering event [9].

A recent study compared postoperative opioid use following posterior spinal fusion surgery (i.e., scoliosis correction surgery) between patients with adolescent idiopathic scoliosis and those with MFS [10]. The results revealed that patients with MFS had greater total morphine consumption, increased use of patient-controlled analgesia, and longer treatment durations. They were also more likely to request additional opioid prescriptions after discharge. These findings raise the question of whether pain in MFS may be partly due to compromised nociception, rather than solely a symptom of skeletal malformations. However, studies of pain in MFS remain scarce and are limited by small sample sizes [11]. Notably, surgical replacement of the aortic root is the gold standard treatment to prevent life-threatening aortic dissection in patients with MFS. Given the high prevalence of chronic pain in this population, careful perioperative management is essential to balance effective analgesia with hemodynamic safety. However, despite these critical considerations, no published studies have systematically assessed postoperative pain intensity or analgesic requirements following aortic-root replacement. Collectively, these gaps highlight the insufficient understanding of pain mechanisms in MFS and the limited efficacy of current therapies. Therefore, it is warranted to develop a suitable animal model to investigate the underlying molecular pathways contributing to nociceptive responses in this syndrome.

Among several mouse models of MFS, the *Fbn1^C1041G/+^* mouse is commonly used to study thoracic aortic aneurysm, as it recapitulates the progressive aortic root dilation and structural abnormalities observed in patients with this syndrome [12]. These mice carry a missense mutation in the *Fbn1* gene, substituting a conserved cysteine with glycine at amino acid 1041 (p.Cys1041Gly). The corresponding residue in human *FBN1* is Cys1039, and mutations at this position (e.g., cysteine substitutions) are associated with classic MFS phenotypes [12,13]. This model has been extensively used to study aortopathy in MFS and to evaluate potential therapies, but studies addressing pain sensitivity have not yet been conducted in these animals. A preclinical study on the onset and progression of pain and muscle deficits associated with MFS could improve our understanding of these symptoms and contribute to the further characterization of the *Fbn1^C1041G/+^* mouse model.

Considering that individuals with MFS may exhibit elevated pain sensitivity and augmented opioid consumption following surgery, we hypothesized that *Fbn1^C1041G/+^* mice, a relevant model for MFS, display heightened pain sensitivity compared to wild-type (WT) mice across sexes. To test this, we assessed pain responses at 2, 4, 6, 8, and 16 months of age using behavioral tests evaluating sensitivity to mechanical and thermal stimuli, as well as grip strength. In addition, we investigated the molecular mechanisms underlying pain in MFS animals through transcriptomic and qRT-PCR analyses of markers associated with inflammation, oxidative stress, excitatory, and nociceptive signaling and chronic pain pathways.

## 2. Materials and Methods

### 2.1. Animals

The *Fbn1^C1041G/+^* mice were purchased from The Jackson Laboratory/Charles River (Lyon, France) and were crossed with WT females from C57BL/6 background. The offspring were then genotyped and categorized as either MFS (*Fbn1^C1041G/+^*) or WT (*Fbn1^+/+^).* Mice were maintained in controlled conditions of temperature (21 ± 1 °C), humidity (66%), and light (12 h light-dark cycle), with food and water available ad libitum. Mice were housed in polypropylene cages with wood shavings, in an enriched environment including a carton tube and cellulose tissues.

A total of 32 mice were used in the RNA-seq study, comprising 16 males and 16 females (*n* = 16 per genotype). Mice aged 3 and 13 months were deeply anesthetized with 5% isoflurane in a 30:70 mixture of O_2_ and N_2_O, and subsequently euthanized.

In a parallel study, a total of 134 mice, 68 males and 66 females, were used to evaluate nociceptive responses using behavioral tests, aortic elastin tears by histology, and the expression of different markers of inflammatory, oxidative, and pain pathways by qRT-PCR assay (Table 1).

The assessment of nociception and muscular deficits was conducted between 9:00 a.m. and 5:00 p.m., following a minimum acclimatization period of seven days to the housing conditions. Animals were also acclimated to the testing room for 1 h before starting the tests. These experiments were conducted in WT and MFS mice of both sexes at 2, 4, 6, 8, and 16 months of age. Mice were euthanized by cervical dislocation, and the aortic tissue and the lumbar section of the spinal cord were extracted at 2, 8, and 16 months of age.

All animal procedures were approved by the local Ethical Committee of Animal Use and Care at the Autonomous University of Barcelona (CEEAH 3041-CEEA-UAB) and by the Generalitat de Catalunya (11517). The procedures were conducted in accordance with the guidelines of the European Communities Council Directive (2010/63/EU) and Spanish Law (RD 53/2013). Every effort was made to reduce the number of animals used and to minimize their distress. To minimize the number of animals used, aortic and spinal cord tissues from animals of 2, 16, and/or 8 months old subjected to the behavioral tests were used to perform the analysis of aortic elastin breaks and qRT-PCR assays.

### 2.2. RNA-Seq

Coronal brain slices from the left hemisphere, covering the striatum and hippocampus, were homogenized in QIAzol lysis reagent (QIAGEN; Hilden, Germany) using a BioRuptor device (Diagenode; Denville, NJ, USA) with five cycles of 30 s on/30 s off. Total RNA was then isolated with the RNeasy Midi Kit (QIAGEN) according to the manufacturer’s instructions. RNA-seq reads were aligned to the *Mus musculus* reference genome (GRCm39) using STAR aligner v2.7.8a [14] under ENCODE-recommended settings. Gene-level quantification followed the approach described in Manich et al., 2025 [15]. Gene set enrichment analysis (GSEA) was performed with the fGSEA R package (v1.12.0), using a pre-ranked gene list (based on the limma moderated t-statistic) tested against mouse REACTOME pathway database. The resulting enrichment profiles were visualized with the ggplot2 package.

### 2.3. Analysis of Elastin Breaks

The ascending thoracic aorta of mice was dissected, free of fat and connective tissue in ice-cold Krebs–Henseleit solution (composition in mM: NaCl 112.0; KCl 4.7; CaCl_2_ 2.5; KH_2_PO_4_ 1.1; MgSO_4_ 1.2; NaHCO_3_ 25.0, and glucose 11.1) gassed with 95% O_2_ and 5% CO_2_. Aortic segments were fixed in 4% phosphate-buffered paraformaldehyde for 1 h, then washed three times with phosphate-buffered saline (PBS). They were incubated overnight in 30% sucrose in PBS, embedded in Tissue-Tek OCT medium (Sakura Finetek Europe, Zoeterwoude, The Netherlands), frozen in liquid nitrogen, and stored at −70 °C. Fourteen-micron aortic sections were stained with an Orcein stain kit (#O1045; Casa Álvarez, Madrid, Spain) to visualize elastic fibers. Slides were examined using an Olympus FV1000 microscope (40× oil immersion objective). At least five representative Orcein-stained images of each mouse aorta were assessed, and a blind observer counted the number of large (≥20 µm) elastin discontinuities. Results were expressed as the number of large discontinuities normalized to the number of elastic laminae.

### 2.4. Nociceptive Behavioral Testing

Mechanical allodynia was assessed with the von Frey (VF) filaments test, measured as the paw withdrawal response to stimulation with different-strength filaments. Mice were placed on an elevated wire grid, in methacrylate cylinders (20 cm tall, 9 cm wide), and left to acclimate for at least 15 min. The different von Frey filaments (North Coast Medical, Inc., San Jose, CA, USA), ranging from 0.4 to 3.5 g of bending force, were each applied to the plantar surface of the hind paws by using the up–down paradigm. The tests began with the weakest filament (0.4 g), and when no reaction was evoked, a filament of greater strength was then used. When a certain filament elicited a positive reaction (hind paw withdrawal), a filament of lesser strength was then used. The mechanical allodynia threshold was determined using an Excel program (Microsoft Iberia SRL, Barcelona, Spain) containing the curve fit of the data.

Thermal hyperalgesia was assessed using the plantar test (PT; Ugo Basile, Varese, Italy), measured as the paw withdrawal latency (s) to stimulation with an infrared heat source. Mice were placed on a glass surface, in polypropylene cylinders (9 cm wide, 20 cm tall), and left to acclimate for 1 h. The infrared heat source was applied to the plantar surface of the hind paws until withdrawal, and three measurements were taken of each paw. To avoid causing damage to the paw, mice were exposed to the heat source for a maximum of 12 s.

Cold allodynia was measured as the paw withdrawal response to a continuous cold stimulus, using the cold plate test (CP; Ugo Basile, Varese, Italy). Each mouse was placed on a CP (5 °C), confined by a polypropylene cylinder, and the number of paw elevations of each hind limb were quantified for 5 min.

In all tests, both hind paws were assessed.

### 2.5. Grip Strength

Grip strength (GS) was determined using a computerized grip strength meter (Model 47200; Ugo Basile; Varese, Italy) which measures the maximal force applied onto a metal bar connected to the recorder. Each mouse was allowed to grasp the metal bar with both its hind legs, while having its upper body supported and its tail gently pulled back by the investigator. The maximum force (g) of the hind legs was recorded, and at least three measurements were taken per mouse.

### 2.6. Analysis of mRNA Levels by Quantitative Real-Time PCR (qRT-PCR)

For gene expression analysis, 2 and 16-month-old mice were euthanized and perfused with sterile saline. A 4 mm segment of the lumbar spinal cord was homogenized using QIAzol lysis reagent (QIAGEN, #R1051). RNA was extracted using the Quick-RNA MiniPrep Kit (Zymo Research, Irvine, CA, USA, #R1055) following the manufacturer protocol. RNA concentration was determined using a NanoDrop spectrophotometer. A total of 650 ng of RNA was reverse-transcribed into cDNA using the High-Capacity cDNA Reverse Transcription Kit from Applied Biosystems (Thermo Fisher Scientific, Waltham, MA, USA, #4368813) according to the manufacturer’s instructions. RT-qPCR reactions were performed in 10 μL volume containing 0.5 μL of cDNA template, 0.5 μL of each primer at 10 μM (Table 2), and 5 μL of iTaq Universal SYBR Green Supermix (Bio-Rad, Hercules, CA, USA, #1725124). Cqs of GAPDH were used to normalize the expression of the target sequences, and relative expression levels were calculated using the 2^−ΔΔCt^ method.

### 2.7. Experimental Procedure

In an initial experiment, we examined the transcriptional changes linked to pain-related signaling pathways in coronal brain sections from the left hemisphere of MFS and WT male and female animals at 3 and 13 months of age, utilizing RNA-seq-based transcriptomics.

In a second experiment, we evaluated the potential development of mechanical allodynia, thermal hyperalgesia, cold allodynia, and muscular deficits in different groups of male and female MFS and WT mice at 2, 4, 6, 8, and 16 months of age. Tests were performed according to this sequence: VF, PT, CP, and GS. In these experiments, mice were acclimated to the environment and equipment -without exposure to the stimuli being studied- on at least three occasions before testing, spread across a minimum of three days. Experiments were conducted by researchers who were blinded to the experimental conditions.

In a parallel study, we evaluated the aortic elastin tears from male and female WT and MFS animals at 2, 8, and 16 months of age through histological analysis. In a concurrent study, we analyzed the potential alterations in mRNA levels of specific markers related to inflammation, oxidative stress, and pain signaling pathways in the spinal cords of 2- and 16-month-old male and female MFS and WT animals using qRT-PCR.

### 2.8. Malondialdehyde (MDA) Concentrations

Plantar skin MDA levels were measured as an indirect index of lipid peroxidation using the thiobarbituric acid reactive substances assay, following the method described by Ohkawa et al. (1979) [16]. Plantar tissue samples were homogenized in PBS at a concentration of 0.1 mg/mL. Aliquots of 50 µL of the homogenate were used for MDA determination by spectrophotometric measurement at 532 nm.

### 2.9. Nitric Oxide (NO) Levels

Plantar skin levels of NO metabolites (nitrites and nitrates) were determined using the colorimetric Griess reaction, as previously described by Green et al. (1982) [17]. Plantar tissue samples were homogenized in PBS at a concentration of 0.1 mg/mL. Aliquots of 50 µL of the homogenate were used for NO metabolite quantification by spectrophotometric measurement at 540 nm.

### 2.10. Statistical Analyses

Statistical analyses were performed using Prism 8.0 (GraphPad Software, San Diego, CA, USA) and SPSS version 28 (IBM Corp., Armonk, NY, USA). Data are presented as mean values ± standard error of the mean (SEM). Assumptions of normality and homogeneity of variance were verified using the Shapiro–Wilk and Bartlett tests, respectively. Differences in elastin fragmentation between WT and MFS mice were analyzed using a three-way ANOVA followed by Sidak’s multiple comparisons test. Differences in nociceptive behaviors, grip strength, and body weight between WT and MFS animals at specific ages (2 and 16 months) were evaluated using a two-way ANOVA with genotype and sex as factors, followed by Sidak’s post hoc test.

To assess the progression of nociceptive behaviors, grip strength, and body weight over time (2, 4, 6, 8, and 16 months), a three-way ANOVA was applied with genotype, sex, and age as factors, followed by one-way ANOVA and Sidak’s multiple comparisons tests to determine the effects of sex and genotype at each age. Variations in mRNA levels between male and female WT and MFS mice at 2 and 16 months were analyzed using two-way ANOVA with genotype and sex as factors, followed by Sidak’s multiple comparisons test. For RNA-seq analyses, statistical testing for differential gene expression included correction for multiple comparisons using the Benjamini–Hochberg false discovery rate. Pathway-level analyses were conducted using GSEA, with enrichment considered at *p* < 0.1.

The value of *p* < 0.05 was considered a statistically significant difference unless otherwise specified.

## 3. Results

### 3.1. Brain Transcriptomic Analysis Reveals Downregulation of Pain-Related Pathways in Young Male and Aged Female MFS Mice and Age-Dependent Reversal in Males

**Figure 1:**
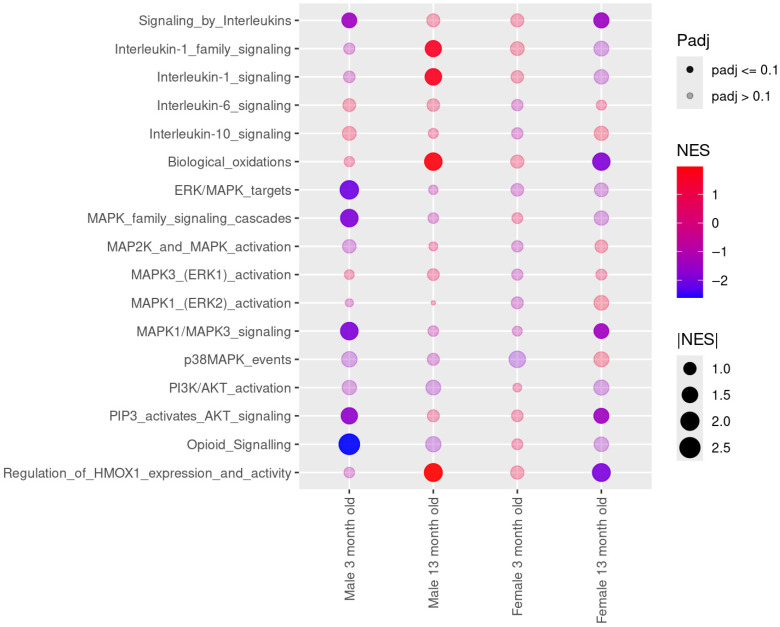
Gene set enrichment analysis (GSEA) in brains from 3- and 13-month-old male and female wild-type (WT) and Marfan syndrome (MFS) mice. Dot plot of combined GSEA results illustrating selected REACTOME inflammation, biological oxidations, excitability and synaptic plasticity, and opioid and heme oxygenase-1 (HMOX1) signaling-related pathways. The color gradient indicates the negative (blue) to positive (red) normalized enrichment score (NES) change, dot size illustrates absolute NES values, and dot transparency indicates whether the result has an adjusted *p*-value (padj) below or equal 0.1. *N* = 4 per experimental group. ERK, extracellular signal-regulated kinase; MAPK, mitogen-activated protein kinase; PI3K, phosphoinositide 3-kinase; AKT, protein kinase B; PIP3, phosphatidylinositol (3,4,5)-trisphosphate.

Firstly, we investigated transcriptional alterations associated with pain-related signaling pathways. RNA-seq transcriptomic analysis was conducted on the brains of young (3-month-old) and aged (13-month-old) male and female WT and MFS mice. Although individual gene-level differential expression analysis revealed limited statistically significant changes, protocadherin gamma subfamily A member 8 (*Pcdhga8*) emerged as the only gene that remained significantly altered after correction for multiple testing based on the false discovery rate in 3-month-old female MFS mice (Supplementary Table S1). In addition, gene set enrichment analysis uncovered pathway-level changes (Figure 1). Several gene sets involved in brain inflammation, neuronal excitability, synaptic plasticity, and opioid signaling—pathways implicated in pain sensitization—were downregulated (*p* < 0.1) at the pathway level in 3-month-old male MFS mice compared with WT mice. In contrast, some of these same pathways remained unaltered in age-matched female MFS mice. Moreover, while in 13-month-old mouse brains, interleukin (IL)-1 signaling, biological oxidations, and heme oxygenase-1 (HMOX1) signaling pathways were upregulated in MFS compared to WT male mice, they were downregulated in aged female MFS mice.

These data suggest a shift in the brain transcriptomic landscape toward reduced activation of pain-related signaling in young male and aged female MFS mice, with an age-dependent reversal observed in aged male MFS mice. To further support these findings, Supplementary Figure S1 shows heatmaps highlighting the log-normalized expression profiles of core enriched genes within opioid and heme oxygenase-1 signaling pathways.

Together, these findings highlight distinct age- and sex-dependent transcriptomic alterations in pain-related signaling pathways in MFS mice, suggesting differential susceptibility to pain modulation across the lifespan and between sexes.

### 3.2. Aortic Elastin Discontinuities Confirm Vascular Pathology in MFS Mice

Because aortic pathology is a hallmark of MFS, we first assessed the extent of aortic elastin breaks in a separate cohort as an indirect marker of vascular damage leading to dilation before evaluating pain-related behaviors. Histological analysis of Orcein-stained sections of the ascending aorta revealed an increased number of large elastin breaks in MFS mice compared to WT controls (Supplementary Figure S2). The two-way ANOVA revealed significant interactions of age and sex (*p* < 0.05), sex and genotype (*p* < 0.05), as well as a main effect of genotype (*p* < 0.001). Indeed, while no statistically significant changes between groups were detected, a significant increase in elastin discontinuities was observed in 8-month-old female MFS mice. Overall, these findings, observed regardless of sex, confirm the presence of aortic pathology in the cohorts.

### 3.3. Mice with MFS Develop Mechanical and Cold Allodynia

**Figure 2:**
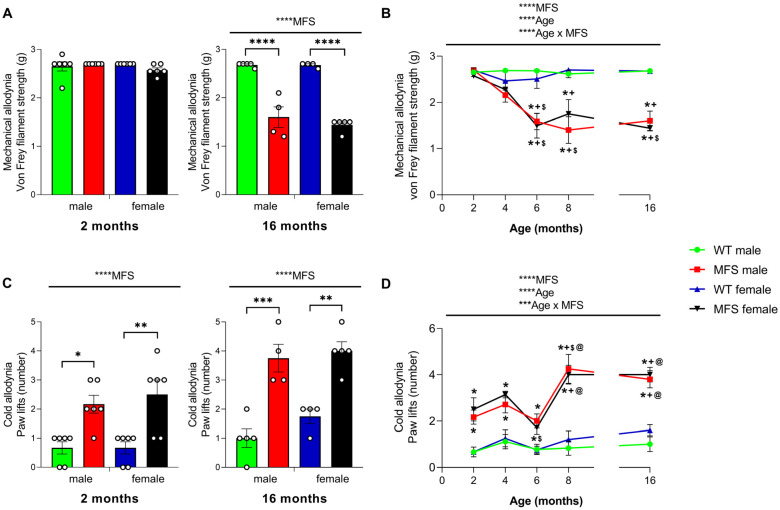
Development of mechanical and cold allodynia in MFS mice**.** Data are presented as the VF filament strength (g) for mechanical allodynia (**A**) and as the number of paw lifts for cold allodynia (**C**) in male and female MFS and WT mice at 2 and 16 months of age. Their respective evolution at different ages is represented in (**B**,**D**). In panels (**A**,**C**), symbols show the degree of statistical significance: * *p* < 0.05, ** *p* < 0.01, *** *p* < 0.001, and **** *p* < 0.0001 (two-way ANOVA followed by Sidak test). In panels (**B**,**D**), symbols above the graphs indicate the level of statistical significance: *** *p* < 0.001 and **** *p* < 0.0001 (three-way ANOVA). Within the graphs, symbols show significant differences: * MFS vs. WT counterpart; + vs. 2 months of age; $ vs. 4 months of age; and @ vs. 6 months of age (*p* < 0.05; one-way ANOVA followed by Sidak test). All data are expressed as mean values ± SEM of at least 4 animals per group.

We evaluated the development of mechanical and cold allodynia in male and female MFS animals at 2, 4, 6, 8, and 16 months of age. In the VF test, the two-way ANOVA showed no significant effects of genotype, sex, or their interaction 2 months-old animals (Figure 2A). However, a significant effect of genotype (*p* < 0.0001) was observed in animals at 16 months. Therefore, while at 2 months, male and female WT and MFS mice had a similar threshold for paw withdrawal in response to VF stimulation, a drastic reduction in the paw withdrawal threshold was observed in MFS mice at 16 months old (*p* < 0.0001 vs. respective WT mice), indicating the development of mechanical allodynia. Regarding the evolution of the mechanical allodynia with age, our results showed that in both male and female MFS mice, the development of mechanical allodynia started at 6 months of age, continued at 8 months, and persisted until 16 months (*p* < 0.0001 vs. 2 months for both sexes and vs. 4 months for females) (Figure 2B). Moreover, in both sexes, the paw withdrawal threshold in MFS mice at 6 months was significantly lower than that at 2 and 4 months (*p* < 0.0392 compared to respective MFS mice), and the threshold at 8 months was also lower than at 2 months (*p* < 0.0107 for both males and females) and at 4 months (*p* < 0.0135 for males). Accordingly, the three-way ANOVA revealed a significant influence of genotype, age, and their interaction (*p* < 0.0001) on the progression of mechanical allodynia over time (Figure 2B).

The two-way ANOVA in the CP test indicated a significant effect of genotype in animals at 2 and 16 months of age (*p* < 0.0001). Therefore, an increased number of paw lifts was observed in MFS female and male mice at both 2 months (*p* < 0.05) and 16 months (*p* < 0.001) in comparison to their corresponding WT mice (Figure 2C), indicating cold allodynia. The analysis of cold allodynia progression in MFS animals (Figure 2D) demonstrated significant effects of genotype and age (*p* < 0.0001) and their interaction (*p* < 0.001), as indicated by the three-way ANOVA. In both male and female MFS animals, the cold allodynia present at 2 months persisted at 4 and 6 months, with the number of paw lifts reaching a zenith at 8 months (*p* < 0.05 compared to 2 months for both sexes, *p* < 0.05 compared to 4 months for males, and *p* < 0.001 compared to 6 months for both sexes) (Figure 2D). This response remained elevated until 16 months of age (*p* < 0.001 for males and *p* < 0.01 for females compared to their respective WT mice), and it was significantly greater than that observed in male and female MFS mice at 2 months (*p* < 0.05) and 6 months of age (*p* < 0.0001) (Figure 2D).

Overall, these findings revealed that MFS mice develop progressive mechanical allodynia starting at 6 months and early-onset cold allodynia from 2 months, with peak severity at 8 months and persistence until at least 16 months.

### 3.4. Mice with MFS Develop Thermal Hyperalgesia in a Sex-Dependent Fashion

We additionally assessed whether MFS was associated with the onset of thermal hyperalgesia in both male and female mice using the PT. The two-way ANOVA demonstrated a significant impact of genotype in both 2-month-old (*p* < 0.0027) and 16-month-old (*p* < 0.0001) subjects.

**Figure 3:**
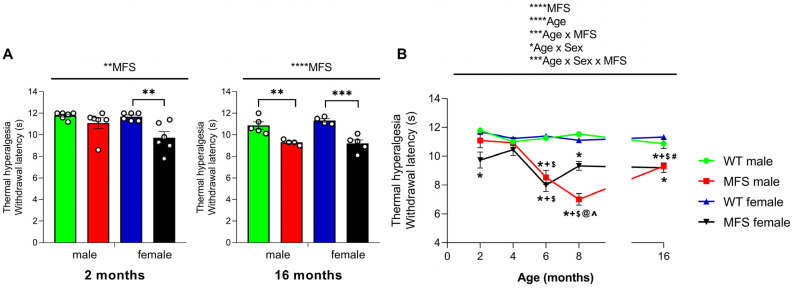
Development of thermal hyperalgesia in MFS mice. Data are presented as the withdrawal latency (s) at 2 and 16 months of age (**A**) and its detailed age evolution (**B**) from male and female MFS and WT mice. In panel (**A**), symbols show the levels of statistical significance: ** *p* < 0.01, *** *p* < 0.001, and **** *p* < 0.0001 (two-way ANOVA with subsequent Sidak test). In panel (**B**), symbols above the graphs indicate the level of statistical significance: * *p* < 0.05, *** *p* < 0.001 and **** *p* < 0.0001 (three-way ANOVA). Within the graphs, symbols indicate statistically significant differences (*p* < 0.05, one-way ANOVA with Sidak post hoc test): * MFS vs. WT counterpart; ^ males vs. female counterpart; + vs. 2 months of age; $ vs. 4 months of age; @ vs. 6 months of age; # vs. 8 months of age. All data are presented as mean values ± SEM of a minimum of 4 animals per group.

This was demonstrated by the lower paw withdrawal latency in response to the infrared heat source observed at 2 months in female MFS mice (*p* < 0.01) and in both male (*p* < 0.01) and female (*p* < 0.001) MFS mice at 16 months, in comparison to their respective WT mice (Figure 3A). In contrast, no differences in paw withdrawal latency were noted between WT and MFS male mice at 2 months (Figure 3A). The data indicated that thermal hyperalgesia manifested earlier in female MFS mice compared to male MFS mice.

Analyzing the progression of thermal hyperalgesia over time, the three-way ANOVA revealed significant effects of genotype (*p* < 0.0001) and age (*p* < 0.0001), as well as interactions between genotype and age (*p* < 0.0001), sex and age (*p* < 0.033), and genotype, sex, and age (*p* < 0.001). Consequently, these data indicated a general reduction in paw withdrawal latency in MFS mice (Figure 3B), reaching a minimum at 6 months in females (*p* < 0.001 vs. 2 and 4 months) and at 8 months in males (*p* < 0.0232 vs. 2, 4, and 6 months). At 8 months, the withdrawal latency observed in MFS male mice was significantly lower than that of their female counterparts (*p* < 0.001). Interestingly, MFS males exhibited a notable enhancement in paw withdrawal latency at 16 months (*p* < 0.0004 vs. 8 months) but still maintained an overall reduced withdrawal latency (*p* < 0.0028 vs. 2 months and *p* < 0.0055 vs. 4 months). Conversely, at 16 months of age, MFS females maintained a decreased withdrawal latency similar to those observed at 8 months. Finally, the withdrawal latency recorded at 6 months in MFS male mice was significantly lower than those observed at 2 and 4 months (*p* < 0.0001).

In summary, thermal hyperalgesia appears earlier in female MFS mice (by 2 months), while males show a delayed but more severe reduction peaking at 8 months, with partial improvement at older ages.

### 3.5. Mice with MFS Lose Grip Strength and Body Weight

**Figure 4:**
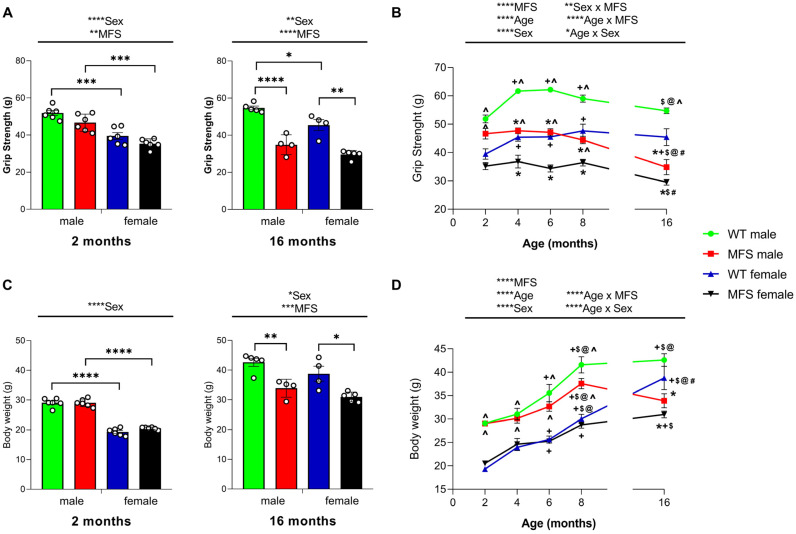
Loss of grip strength and body weight in MFS mice. Data are presented as the grip strength (g) (**A**) and body weight (g) (**C**) in male and female MFS and WT mice at 2 and 16 months of age. Their respective evolution at different ages is represented in (**B**,**D**). In panels (**A**,**C**), symbols show the degree of statistical significance; * *p* < 0.05, ** *p* < 0.01, *** *p* < 0.001, and **** *p* < 0.0001 (two-way ANOVA followed by Sidak test). In panels (**B**,**D**), symbols above the graphs indicate the level of statistical significance: * *p* < 0.05, ** *p* < 0.01, and **** *p* < 0.0001 (three-way ANOVA). Within the graphs, symbols show significant differences; * MFS vs. WT counterpart; ^ males vs. female counterpart; + vs. 2 months of age; $ vs. 4 months of age; @ vs. 6 months of age and # vs. 8 months of age (*p* < 0.05; one-way ANOVA followed by Sidak test). All data are expressed as mean values ± SEM of at least 4 animals per group.

We assessed potential muscle weakness associated with MFS by measuring hindlimb grip strength in male and female mice. Significant effects of sex were revealed by the two-way ANOVA at both 2 (*p* < 0.0001) and 16 months of age (*p* < 0.0021) as well as of genotype at both 2 (*p* < 0.008) and 16 months of age (*p* < 0.0001). Notably, although WT and MFS mice of both sexes exhibited comparable grip strengths at 2 months, a significant decrease in grip strength was observed at 16 months in MFS mice as compared to their respective WT mice (*p* < 0.0001 for males and *p* < 0.0001 for females) (Figure 4A). At both ages, the grip strength of male WT mice was higher than in females (*p* < 0.001 at 2 months and *p* < 0.05 at 16 months), while only at 2 months was the grip strength of male MFS higher than of MFS female mice (*p* < 0.001). The three-way ANOVA analyzing the evolution of grip strength over time revealed significant effects of genotype (*p* < 0.0001), sex (*p* < 0.0001), and age (*p* < 0.0001), along with interactions between genotype and age (*p* < 0.0001), sex and age (*p* < 0.011), and sex and genotype (*p* < 0.008). Therefore, WT mice showed a progressive increase in grip strength as they aged. For example, at 8 months of age, the grip strength was higher than at 2 months (*p* < 0.01 for males and *p* < 0.01 for females) (Figure 4B). However, this pattern was not observed in MFS mice. Instead, the grip strength of both male and female MFS mice remained stable or exhibited a gradual decline with age, reaching the weakest levels at 16 months (*p* < 0.05 vs. 4 and 8 months for females, and *p* < 0.01 vs. 2, 4, 6, and 8 months for males) (Figure 4B). As a result, MFS mice exhibited lower grip strength than WT mice between 4 and 16 months of age (*p* < 0.0001 for males and *p* < 0.001 for females). Furthermore, female mice showed lower grip strength than their male counterparts from 2 to 16 months in WT animals and from 2 to 6 months in MFS animals.

Regarding body weight, the two-way ANOVA revealed an effect of sex in 2-month-old animals (*p* < 0.0001), while in 16-month-old animals, both sex (*p* < 0.0474) and genotype (*p* < 0.0001) demonstrated significant effects. Therefore, while WT and MFS males had a larger body weight than their respective females at 2 months (*p* < 0.0001), at 16 months, WT and MFS female mice showed lower body weight as compared to their respective males, but it was not significant statistically (Figure 4C). Nevertheless, at 16 months, both male and female MFS mice exhibited reduced body weight relative to their corresponding WT mice (*p* < 0.050). Analyzing the progression of total body weight, the three-way ANOVA revealed significant effects of genotype (*p* < 0.0001), sex (*p* < 0.0001) and age (*p* < 0.0001), as well as the interactions between genotype and age (*p* < 0.0001), and sex and age (*p* < 0.001). Therefore, WT mice showed a progressive increase in body weight as they aged; for instance, at 8 months, the body weight of male and female WT mice was higher than their respective weights at 2, 4, and 6 months (*p* < 0.001) (Figure 4D). However, this pattern was only observed in MFS male mice, whose body weight increased with age, reaching a peak at 8 months, surpassing the weights recorded at 2, 4, and 6 months (*p* < 0.05). MFS females experienced the greatest increase in body weight at 16 months, which was higher than at 2 and 6 months of age (*p* < 0.001). Nevertheless, while the body weight in WT increased until 16 months in males (*p* < 0.001 vs. 2, 4, and 6 months) and females (*p* < 0.0001 vs. 2, 4, 6, and 8 months), it slowly decreased until 16 months in MFS male mice. As a result, the body weight in MFS mice was lower than in WT mice at 16 months of age (*p* < 0.0306). At this age, the body weight of female MFS mice was also lower that their respective WT mice (*p* < 0.0194). As observed with grip strength, male animals of both genotypes exhibit greater body weight than females from 2 to 8 months (*p* < 0.050).

In summary, WT mice showed progressive increase in grip strength and body weight with age, whereas MFS mice exhibited impaired grip strength and reduced body weight, particularly evident at 16 months. Male mice generally had greater grip strength and body weight than females across both genotypes.

### 3.6. Effect of MFS on Spinal Cord Inflammatory Signaling Pathways Involved in Pain Modulation

To address the potential mechanisms underlying the altered nociceptive responses observed in MFS mice, we first evaluated the mRNA levels of several inflammatory mediators, such as *Il6*, *Tnf* and *Nos2* in the spinal cord of these animals at 2 and 16 months of age.

**Figure 5:**
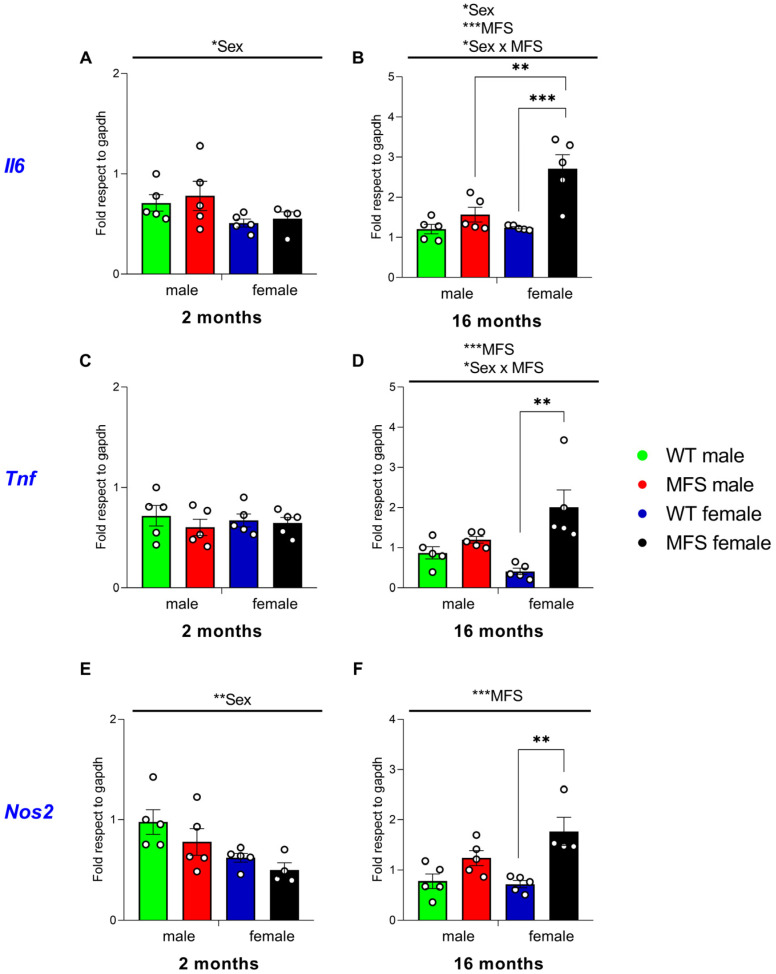
Expression of *Il6*, *Tnf* and *Nos2* in the spinal cord of MFS mice at 2 and 16 months of age. Data are presented as fold respect to GAPDH for *Il6* (**A**,**B**), *Tnf* (**C**,**D**), and *Nos2* (**E**,**F**) in the spinal cord of male and female WT and MFS animals at 2 and 16 months of age. In all panels, symbols show the level of statistical significance; * *p* < 0.05, ** *p* < 0.01, and *** *p* < 0.001 (one-way ANOVA followed by Sidak test). Data are expressed as mean values ± SEM; *n* = 4–5 samples per group.

Regarding *Il6*, the two-way ANOVA revealed significant effect of sex at 2 months of age (*p* < 0.0407), while at 16 months a significant effect of genotype (*p* < 0.0004), sex (*p* < 0.0109), and their interaction (*p* < 0.0156) were demonstrated. Then, while no changes between groups were detected at 2 months (Figure 5A), a significant increased expression of Il6 was observed in the spinal cord of 16-month-old female MFS mice (*p* < 0.0007 vs. their respective WT females) (Figure 5B). Moreover, at 16 months old, female MFS showed higher *Il6* expression compared to male MFS (*p* < 0.0069) (Figure 5B). No changes in the expression of this inflammatory marker were detected when comparing male MFS and WT mice at 2 and 16 months of age.

With respect to *Tnf*, while at 2 months no changes were detected by the two-way ANOVA, a significant effect of genotype (*p* < 0.0009) and its interaction with sex (*p* < 0.0162) were observed in animals of 16 months of age. Indeed, a significant increase in the expression of *Tnf* was manifested in 16-month-old MFS females compared to their 16-month-old WT counterparts (*p* < 0.0013) (Figure 5D). No alterations in the expression of this protein were observed between male MFS and WT mice of 2 (Figure 5C) or 16 months of age.

As for *Il6* and *Tnf*, the expression of *Nos2* was also markedly elevated in female MFS mice compared to their corresponding WT counterparts at 16 months of age (*p* < 0.003) (Figure 5F), although this difference was not evident at 2 months (Figure 5E). The two-way ANOVA conducted on 16-month-old subjects validated these findings by indicating a significant effect of genotype (*p* < 0.0003). In animals of 2 months old, the two-way ANOVA also revealed a significant effect of sex (*p* < 0.0078) on the *Nos2* expression.

Overall, these results indicate that the increased spinal cord expression of pro-inflammatory mediators (*Il6*, *Tnf*, and *Nos2*) in MFS mice is age- and sex-dependent, and it is particularly noticeable in 16-month-old female mice.

### 3.7. Effect of MFS on Spinal Cord Oxidative Stress Signaling Pathways Involved in Pain Modulation

**Figure 6:**
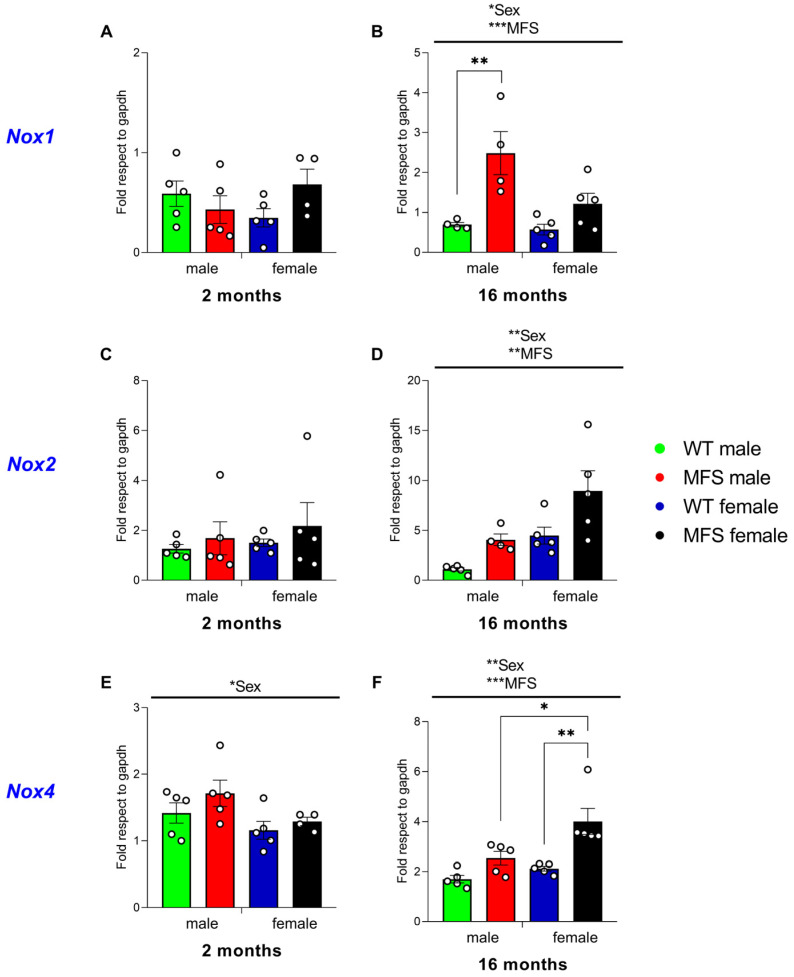
Expression of *Nox1*, *Nox2*, and *Nox4* in the spinal cord of MFS mice at 2 and 16 months of age. Data are presented as fold respect to GAPDH for *Nox1* (**A**,**B**), *Nox2* (**C**,**D**), and *Nox4* (**E**,**F**) in the spinal cord of male and female WT and MFS mice at 2 and 16 months of age. In all panels, symbols show the degree of statistical significance; * *p* < 0.05, ** *p* < 0.01, and *** *p* < 0.001 (one-way ANOVA followed by Sidak test). Data are expressed as mean values ± SEM; *n* = 4–5 samples per group.

Focusing on oxidative stress, the expression of *Nox1*, *Nox2*, and *Nox4* in the spinal cord of MFS and WT male and female mice of 2 and 16 months of age was assessed. For *Nox1*, the two-way ANOVA only revealed a significant effect of sex (*p* < 0.0317) and genotype (*p* < 0.0009) in 16-month-old animals. No variations in the expression of this oxidative stress marker were detected among MFS and WT mice of both sexes at 2 months of age (Figure 6A). Conversely, a marked elevation in *Nox1* expression was noted in male MFS mice aged 16 months compared to their WT male counterparts (*p* < 0.0063) (Figure 6B), whereas the mRNA levels of *Nox1* in 16-month-old MFS female mice was unchanged relative to their 16-month-old WT counterparts.

The two-way ANOVA demonstrated significant effects of sex (*p* < 0.0034) and genotype (*p* < 0.0069) on the mRNA levels of *Nox2* in 16-month-old MFS and WT animals (Figure 6D). No significant differences were observed among MFS and WT mice at 2 (Figure 6C) or 16 months of age (Figure 6D).

In addition, *Nox4*, in contrast to *Nox1* and *Nox2*, old MFS females exhibited significant higher levels of this oxidative stress marker than their respective WT counterparts (*p* < 0.0029). In contrast, no relevant alterations in *Nox4* expression were detected in male MFS and WT mice of 2 or 16 months of age, and none when comparing 2-month-old female MFS mice with their corresponding WT counterparts (Figure 6E). These findings were corroborated by the significant effects of sex (*p* < 0.0072) and genotype (*p* < 0.0004) indicated by the two-way ANOVA in 16-month-old animals (Figure 6F). Moreover, at 16 months old, female MFS showed higher *Nox4* expression compared to male MFS (*p* < 0.0220) (Figure 6F).

Taken together, these findings indicate an age- and sex-dependent increased expression of oxidative stress markers in the spinal cord of MFS mice, with *Nox1* elevated in 16-month-old males and *Nox4* increased in 16-month-old females.

### 3.8. Effect of MFS on the Expression of Grin1, Gria1, Gria2, and Trpv1 in the Spinal Cord

**Figure 7:**
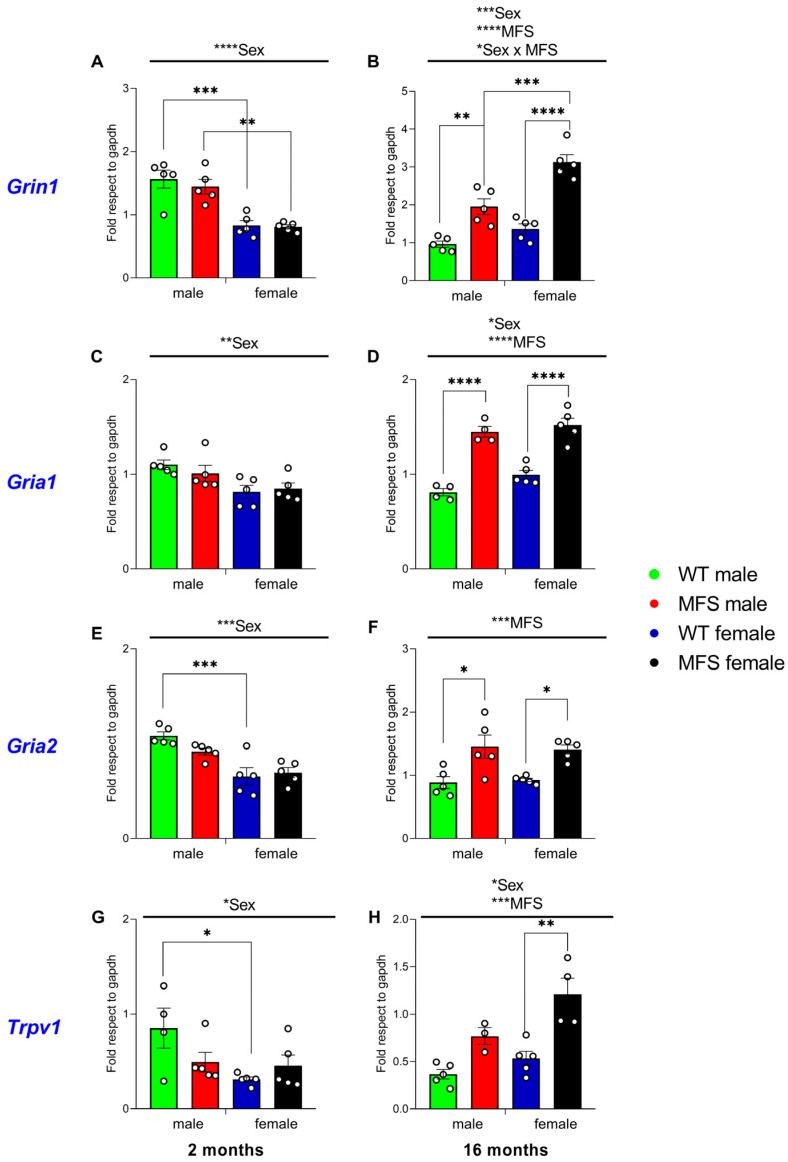
Expression of *Grin1*, *Gria1*, *Gria2*, and *Trpv1* in the spinal cord of MFS mice at 2 and 16 months of age. Data are presented as fold respect to GAPDH for *Grin1* (**A**,**B**), *Gria1* (**C**,**D**), *Gria2* (**E**,**F**), and *Trpv1* (**G**,**H**) in the spinal cord of male and female WT and MFS mice at 2 and 16 months of age. In all panels, symbols show the degree of statistical significance; * *p* < 0.05, ** *p* < 0.01, *** *p* < 0.001 and **** *p* < 0.0001 (one-way ANOVA followed by Sidak test). Data are expressed as mean values ± SEM; *n* = 4–5 samples per group.

The two-way ANOVA showed a significant effect of sex (*p* < 0.0001) on *Grin1* (N-methyl-D-aspartate, NMDA, receptor subunit) expression at 2 months of age. At 16 months, significant effects of genotype (*p* < 0.0001), sex (*p* < 0.0002), and their interaction (*p* < 0.0300) were observed. Accordingly, at 2 months, *Grin1* expression differed significantly between WT (*p* < 0.0006) and MFS (*p* < 0.0024) animals in male vs. female mice (Figure 7A). At 16 months, significant differences were detected between male MFS and WT mice (*p* < 0.0031) and between female MFS and WT mice (*p* < 0.0001), as well as between male and female MFS mice (*p* < 0.0006) (Figure 7B).

Concerning *Gria1* (α-amino-3-hydroxy-5-methyl-4-isoxazolepropionic acid, AMPA, receptor subunit), the two-way ANOVA revealed a significant effect of sex at 2 months of age (*p* < 0.0039). At 16 months, significant effects of sex (*p* < 0.0453) and genotype (*p* < 0.0001) were observed. Accordingly, no significant genotype-specific differences were detected among 2-month-old mice (Figure 7C), whereas at 16 months, a significant increase in *Gria1* expression was observed in both male (*p* < 0.0001) and female (*p* < 0.0001) MFS mice compared with their respective WT controls (Figure 7D).

*Gria2* expression was also evaluated. The two-way ANOVA revealed a significant effect of sex in 2-month-old mice (*p* < 0.0001) and a significant effect of genotype in 16-month-old mice (*p* < 0.0002). Accordingly, at 2 months of age, a significant decreased expression of *Gria2* expression was observed in WT female mice compared with their male counterparts (*p* < 0.0007) (Figure 7E). In contrast, at 16 months, *Gria2* expression was significantly increased in both male (*p* < 0.0127) and female (*p* < 0.0410) MFS mice compared with their respective WT controls (Figure 7F).

Finally, *Trpv1* expression was evaluated in the spinal cord of WT and MFS mice of both sexes. The two-way ANOVA revealed a significant effect of sex at 2 months (*p* < 0.0297) and at 16 months of age (*p* < 0.0111), as well as a significant effect of genotype (*p* < 0.0002) at 16 months. Accordingly, at 2 months of age, a significant decrease in *Trpv1* expression was observed in WT female mice compared with their male counterparts (*p* < 0.0453) (Figure 7G). In contrast, at 16 months, *Trpv1* expression was significantly increased in MFS female mice compared with their respective WT female controls (*p* < 0.0019) (Figure 7H).

Overall, the expression of *Grin1*, *Gria1*, *Gria2*, and *Trpv1* in the spinal cord was modulated by age, sex, and genotype. Sex-dependent differences were evident at early ages, whereas genotype-dependent changes were mainly detected in MFS mice at 16 months. These effects were particularly pronounced for *Grin1*, *Gria1*, and *Gria2* in both sexes, while *Trpv1* upregulation was restricted to older female MFS mice.

### 3.9. Effect of MFS on Other Spinal Cord Signaling Pathways Involved in Pain Modulation

We evaluated the effects of MFS on the expression of *Tgfb1*, *Hmox1*, and *Oprm1* in the spinal cord. *Tgfb1* was selected as a context-appropriate TGFβ-related gene to explore pain-associated signaling pathways in our central nervous system-focused analyses [18]. *Hmox1* was included because of its contribution to antioxidant and antinociceptive responses at the spinal level [19]. *Oprm1* was chosen because the μ-opioid receptor, encoded by this gene, is essential for analgesia and is expressed in both central and peripheral nervous system [20].The two-way ANOVA demonstrated a significant effect of genotype (*p* < 0.0154) on *Tgfb1* expression at early ages (2 months) and significant effects of genotype (*p* < 0.0001) and its interaction with sex (*p* < 0.0473) on *Tgfb1* expression at older ages (16 months). Consequently, while no significant differences were identified between 2-month-old male or female MFS mice and their corresponding WT counterparts (Supplementary Figure S3A), significant differences were noted between 16-month-old MFS and WT males (*p* < 0.0006) (Supplementary Figure S3B). Conversely, comparable Tgfb1 levels were noted between 16-month-old MFS and WT females.

Concerning *Hmox1*, while the two-way ANOVA indicated a significant sex effect at 2 months (*p* < 0.0043) and 16 months (*p* < 0.0087), no significant differences in the expression of this antioxidant enzyme were observed when comparing male and female MFS and WT mice at either 2 months (Supplementary Figure S3C) or 16 months of age (Supplementary Figure S3D).

Finally, the expression of *Oprm1* was also evaluated, revealing a significant effect of sex (*p* < 0.0446) in 2-month-old animals, while a notable interaction between sex and genotype (*p* < 0.0046) was observed in 16- month-old animals. Consequently, while the expression of *Oprm1* remained unchanged in male and female MFS and WT mice at 2 months of age (Supplementary Figure S3E) and in 16-month-old males of both genotypes, a reduction in the expression of this receptor was noted in female MFS mice at 16 months compared to their WT female counterparts (*p* < 0.0144) (Supplementary Figure S3F).

These results reveal a complex, age- and sex-specific pattern in the expression of key signaling pathways involved in pain modulation in MFS mice. *Tgfb1* expression is upregulated in aged MFS males, *Hmox1* remains largely unchanged across groups, and *Oprm1* is selectively reduced in aged MFS females.

### 3.10. Peripheral Plantar Oxidative and Nitrosative Markers Are Not Altered in Aged MFS Mice

To assess whether peripheral oxidative or nitrosative stress differed across groups, MDA levels and NO metabolites were measured in plantar skin from 16-month-old WT and MFS mice of both sexes. As shown in Supplementary Figure S4, no significant differences were observed in MDA or NO_x_ levels as a function of genotype or sex. These results suggest that peripheral plantar oxidative and nitrosative status is preserved in aged MFS mice.

## 4. Discussion

Pain is a prevalent and incapacitating condition in individuals with MFS. However, there is a lack of comprehensive understanding regarding this symptomatology and the main mechanisms contributing to its onset and progression with age. In this investigation, we assessed the potential development of nociceptive responses in both male and female *Fbn1^C1041G/+^* mice, a relevant model for MFS, and investigated the main molecular pathways involved.

The transcriptomic analysis of the brains of WT and MFS mice identified distinct age- and sex-specific alterations in pain-related signaling in MFS mice. Consistent with previous reports describing sex- and age-dependent neurovascular and neuroinflammatory alterations in MFS [15,21], we observed a downregulation of pathways associated with neuroinflammation, neuronal excitability, synaptic plasticity, and opioid signaling in young male and aged female MFS mice, suggesting a reduced activation of central pain-sensitization mechanisms in these groups. Interestingly, this pattern is reversed in aged male MFS mice, where pro-inflammatory and oxidative stress-related pathways, including IL-1 signaling and HMOX1 are upregulated in line with prior evidence that IL-1β and HMOX1 are induced in aged male brains [22,23]. These transcriptomic shifts may indicate age-related adaptations in pain processing, which differ between sexes. In particular, the changes observed in young males and aged females could reflect compensatory modulation of pain-related pathways, while the reversal seen in aged males points to a transcriptomic re-activation over time. The lack of major pathway alterations in young female MFS mice further highlights sex-specific patterns in central pain regulation, in agreement with prior observations that nociceptive and opioid signaling mechanisms show strong sex dependence [24,25]. These findings suggest that pain-related brain transcriptomic signaling is altered in MFS mice and emphasize the influence of both age and sex.

In light of these molecular changes in the brain, we examined whether age- and sex-dependent differences were also reflected at the behavioral level by assessing the possible development of allodynia and hyperalgesia in young (2-month-old) and adult (16-month-old) male and female MFS mice. Our results show that at 2 months of age, MFS mice of both sexes manifested cold allodynia, whereas thermal hyperalgesia was observed only in females, highlighting a sex-specific effect on early pain development. Interestingly, cold allodynia appeared as the earliest nociceptive response at 2 months, whereas thermal hyperalgesia and mechanical allodynia required 6 months to become established, indicating that the onset of these symptoms is age-dependent. By 16 months of age, MFS mice of both sexes exhibited mechanical and cold allodynia, thermal hyperalgesia, and reduced grip strength, suggesting that nociceptive and motor deficits progress with age. These findings indicate that *Fbn1^C1041G/+^* mice recapitulate the pain hypersensitivity observed in MFS patients, as reflected by the higher and more frequent opioid doses required for recovery following scoliosis correction surgery [10], mirror the tendency for pain to be more severe in older individuals with MFS [7,8], and further contribute to the characterization of this model for studying MFS-related nociceptive mechanisms. Of note, the prevalence of pain in MFS patients varies widely [5]. This variability is expected, as over 2000 mutations have been identified in the *FBN1* gene, most of which are unique and family-specific [3]. Notably, the p.Cys1041Gly mutation in *Fbn1* (present in the *Fbn1^C1041G/+^* mouse) affects the same calcium-binding EGF-like (cbEGF) domain as a well-characterized human missense mutation, p.Cys1039Tyr [12], reinforcing the clinical relevance of our findings.

At 2 months, when aortic aneurysms are already present in *Fbn1^C1041G/+^* mice [12], both sexes manifested only cold allodynia. This suggests that beyond cold allodynia, other nociceptive responses may emerge as molecular changes accompany aortic deterioration. Cold allodynia may therefore represent a relevant clinical symptom to consider in MFS, because although not specific to the disease, it appears linked to its progression. Our data further revealed sex-specific patterns in hyperalgesia. In MFS females, hyperalgesia was already evident at 2 months and worsened slightly with age. In contrast, MFS males showed no hyperalgesia at 2 months, but it became more pronounced at 8 months and eventually reached levels similar to females by 16 months. Women are more likely to report pain and chronic pain than men [26]. For example, in C57BL6/J mice after spinal cord injury, females displayed greater mechanical and thermal hypersensitivity than males [27]. Likewise, in a survey of MFS patients, women reported pain more often than men (77% vs. 70%) [28]. Although pain is generally more pronounced in females, other MFS traits such as aortic aneurysm tend to be more severe in males. Future studies should therefore investigate how aortic pathology interacts with pain mechanisms in MFS.

Previous evidence has shown that musculoskeletal manifestations in MFS worsen with age, including decreases in bone mineral content and muscle mass [3,29]. In accordance, our study reveals a halt in strength gain in MFS mice compared to WT mice, followed by a gradual decline with age. At 2 months, no differences in grip strength were detected between MFS and WT mice. However, from 4 months onward, MFS mice showed a significant decrease in grip strength compared to WT animals. At 16 months, sex-specific differences between genotypes became statistically significant: grip strength was lower in female than in male mice, in both genotypes. These findings indicate that significant muscular deficits in MFS emerge primarily at advanced ages, with females showing greater vulnerability. In accordance with these results, muscular deficiencies have also been observed in animals with chemotherapy-induced neuropathic pain, where female mice displayed lower grip strength than males [30]. This further suggests that sex influences susceptibility to muscle weakness.

A similar pattern was observed in body weight, with MFS mice showing lower values than sex-matched WT mice at 16 months, which may contribute to the muscular weakness observed in older animals. Overall, these findings highlight muscular deficiencies in MFS mice, further validating the *Fbn1^C1041G/+^* strain as a suitable preclinical model to study the onset and progression of pain and muscle weakness in this syndrome.

We analyzed the spinal cord expression of key excitatory and nociceptive receptor subunits, including *Grin1*, encoding the obligatory subunit of the N-methyl-D-aspartate (NMDA) receptor, *Gria1* and *Gria2*, which encode subunits of the α-amino-3-hydroxy-5-methyl-4-isoxazolepropionic acid (AMPA) receptor, and *Trpv1*, encoding the transient receptor potential vanilloid 1 (TRPV1) channel. Notably, all four transcripts were consistently upregulated in the spinal cord of 16-month-old MFS mice compared with WT animals, independently of sex (Figure 7). Given the well-established role of NMDA and AMPA receptors in central sensitization and synaptic plasticity associated with chronic pain [31,32], together with the contribution of TRPV1 to thermal and mechanical hypersensitivity [33], these transcriptional changes are fully consistent with the pronounced pain hypersensitivity observed behaviorally in aged MFS mice. Collectively, these findings further support the notion that enhanced excitatory neurotransmission and nociceptive signaling at the spinal level contribute to the maintenance of chronic pain in MFS.

Moreover, although the direct mechanisms linking fibrillin-1 deficiency to neuronal signaling remain incompletely defined, altered extracellular matrix architecture in MFS may modify cell–matrix interactions, including integrin-mediated signaling and mechanotransduction [34], and compromise neurovascular unit integrity [35]. These changes may, in turn, influence neuronal excitability and sensory processing, including pain-related pathways.

Cytokines mediate neuroinflammation and are involved in the development of chronic pain. Recent studies have found that sex is involved in the regulation of pain thresholds by ILs [36]. In this study, we evaluated the expression of *Il6* and *Tnf* in the spinal cord of MFS animals at 2 and 16 months old. In accordance with other preclinical models of chronic pain, an increased expression of *Il6* and *Tnf* was detected in the spinal cord of female MFS at 16 months as compared to their respective female WT mice of 16 months. These findings parallel the heightened neuroinflammatory signaling identified in aged female brains, suggesting that brain and spinal mechanisms may act synergistically to drive enhanced pain sensitivity in this group. Considering the nociceptive role played by cytokines in chronic pain, the increased levels of *Il6* and *Tnf* detected in female MFS animals at 16 months might be explained, at least in part, by the mechanical and cold allodynia and the heightened sensitivity to heat stimulus observed in MFS females at this age. In contrast to females, no changes in the expression of these cytokines were observed in the spinal cord of MFS male at 16 mounts old, thus revealing that the MFS-associated increased expression of these cytokines differed among sexes. In line with our findings, several sex differences in the expression of ILs and of the NLRP3 inflammasome, which is responsible for IL-1β maturation and release in the spinal cord has been reported. Specifically, IL-1β mRNA levels are higher in females than in males following nerve injury, and NLRP3 inflammasome is more highly expressed in females than male mice [37]. Similarly, the expression of *Nos2* is also increased in the spinal cord of MFS female mice at 16 months old, but not in males. Increased expression of this inflammatory mediator has also been observed in the spinal cord of animals with neuropathic pain, and *Nos2*-KO mice fail to develop allodynia and hyperalgesia after nerve injury [38]. It is also well established that *Nos2* plays a major role in thoracic aortic aneurysm, as both genetic and pharmacological studies demonstrate its involvement in aortic dilation and medial degeneration in MFS [39].

Recent studies indicate that the roles and mechanisms of action of some ILs in chronic pain differ between sexes and that the etiology of pain can further modulate their effects [36]. Consistently, our data show that at 16 months of age, the increased expression of *Il6* and *Tnf* in MFS mice compared with WT was more pronounced in females. Interestingly, elevated IL-6 has also been implicated in muscle atrophy, and its inhibition might reverse this effect [40]. Nevertheless, considering that losing grip strength is the only thing that seems equal in male and female MFS mice as compared to WT, and IL6 is increased only in females, we hypothesized that MFS mice at 16 months old experience an enhanced aging phenotype that might be associated with age-related neuromuscular decline, potentially involving mitochondrial dysfunction and altered metabolic regulation [41,42].

In addition, reactive oxygen species are key mediators in chronic pain and that NADPH oxidases (*Nox1*, *Nox2*, and *Nox4*) play central roles. In our study, 16-month-old MFS males showed elevated spinal mRNA levels of *Nox1*, while females showed higher *Nox4* expression compared to WT counterparts; no changes were observed at 2 months. These findings suggest that oxidative stress contributes to the maintenance of chronic pain in aged MFS mice, with sex-dependent involvement of distinct NOX isoforms (*Nox1* in males, *Nox4* in females). This is in accordance with previous evidence showing upregulation of these enzymes in experimental pain models and beneficial effects of their deletion or inhibition [43,44,45]. Notably, the increased *Nox1* expression in MFS males may help explain the higher opioid requirements reported in MFS patients, while females exhibited concurrent increases in *Nox4*, *Il6*, *Tnf*, and *Nos2*, indicating stronger oxidative and inflammatory responses. Of note, *Nox4* upregulation appears to be a hallmark molecular feature of MFS, having been reported in the brain of both sexes [15], in the male aorta [46], and in male cerebral arteries [47]. This systemic pattern, together with the concurrent increases in *Il6*, *Tnf*, and *Nos2* in females, underscores stronger oxidative and inflammatory responses and may help explain sex-specific differences in pain sensitivity and opioid requirements in MFS. Notably, peripheral oxidative and nitrosative markers in plantar skin were unchanged across sex and genotype in aged mice, supporting a predominant contribution of central mechanisms to the pain phenotype observed in MFS.

The TGF-β signaling cascade has a complex and often contradictory role, acting as both pro-nociceptive and antinociceptive depending on the pathophysiological context. For example, TGF-β1 induces neuronal hyperexcitability and contributes to chronic pain during bone cancer and pancreatitis [48,49], but its levels might be low in subjects with osteoarthritis or neuropathic pain, suggesting antinociceptive or protective effects through inhibition of neuroimmune responses and promotion of endogenous opioid expression in the spinal cord [48,50,51]. Such discrepancies likely reflect activation of distinct receptor-mediated pathways and the balance between central vs. peripheral actions of this cytokine [52,53]. In our study, we detected an upregulation of TGF-β1 mRNA in the spinal cord of 16-month-old male MFS mice, a change absent in females and in younger animals. This finding is particularly relevant considering the divergent roles reported for TGF-β1 in pain modulation. In the context of MFS, where excessive TGF-β signaling is also linked to aortic pathology [3], enhanced spinal *Tgfb1* expression in aged males may contribute to their increased pain sensitivity, potentially through mechanisms distinct from those operating peripherally. Importantly, this upregulation complements the pro-inflammatory transcriptomic profile observed in the brains of aged males, highlighting coordinated yet region-specific mechanisms in pain sensitization. Similarly, the downregulation of Orpm1 in the spinal cord of aged females may reflect compensatory or maladaptive processes not evident at the brain level, emphasizing that different regions of the central nervous system contribute distinctively to the pain phenotype. Overall, these observations underscore the need for a systematic dissection of the context- and sex-specific roles of TGF-β in central pain modulation.

The antioxidant system modulates pain and HMOX1 inducers produce analgesic effects by reducing oxidative stress [54,55]. In our study, spinal cord *Hmox1* mRNA expression in MFS mice did not differ from WT mice at any age, in agreement with previous observations in neuropathic pain and type 1 diabetes models [56,57], suggesting that changes in the protein levels may occur in the brain rather than in the spinal cord of MFS adult mice. Interestingly, while aged male MFS brains exhibited upregulation of oxidative stress-related pathways including *Hmox1*, in the spinal cord we observed a sex-specific upregulation of Nox isoforms. These findings suggest that although both regions contribute to redox imbalance, distinct molecular mediators may operate in a region- and sex-specific manner to maintain chronic pain. In addition, the opioid system is key for pain regulation, we observed reduced *Orpm1* mRNA in the spinal cord of 16-month-old female MFS mice, consistent with µ-opioid receptor downregulation following nerve injury [58,59,60] and with higher opioid use in MFS patients [10]. In contrast, male MFS mice showed no spinal *Orpm1* changes despite similar hyperalgesia, highlighting sex-specific differences and suggesting receptor downregulation develops later in the disease. Future studies should examine the temporal progression of *Orpm1* expression and the influence of sex hormones in MFS-associated pain.

This study has several limitations: (i) While this study identifies robust nociceptive phenotypes, the relationship between aortic pathology severity and pain hypersensitivity was not directly examined and warrants further investigation. (ii) Cytokine- and signaling-related changes were primarily assessed at the mRNA level, which may not fully reflect protein expression or functional activity. (iii) Comparisons were performed between age groups rather than through longitudinal follow-up of the same animals, limiting conclusions regarding temporal progression. (iv) Although age- and sex-dependent molecular signatures associated with pain hypersensitivity were identified, the lack of cell-type-specific analyses in key nociceptive structures, such as the dorsal root ganglia and somatosensory cortex, limits mechanistic resolution. Future studies employing targeted and cell-specific approaches will be essential to directly link transcriptomic alterations to neural circuit dysfunction and behavioral outcomes. (v) As this work was conducted in a specific murine model of MFS, the generalizability of the findings to other models and to human disease warrants caution.

## 5. Conclusions

In conclusion, this study provides the first preclinical demonstration of pain development in a mouse model of MFS. We show that *Fbn1^C1041G/+^* mice develop progressive pain sensitivity, characterized by allodynia and hyperalgesia that worsen with age and display sex-dependent patterns. These animals also display the characteristic muscular weakness that mirrors clinical observations in MFS patients. Our findings not only implicate inflammation and oxidative stress as key molecular contributors to pain and muscle decline but also suggest a novel link between MFS and cold allodynia. Importantly, the absence of peripheral oxidative alterations, along with marked changes in spinal excitatory and nociceptive markers, suggests a predominant role of central mechanisms in the MFS pain phenotype. Together, these insights expand the current understanding of MFS beyond cardiovascular manifestations and position pain as a significant clinical feature of the disease. The *Fbn1^C1041G/+^* mouse emerges as a powerful and translationally relevant preclinical model, offering unique opportunities for dissecting underlying mechanisms and developing targeted therapies aimed at improving the quality of life of individuals with MFS.

## Figures and Tables

**Table 1:** Number of mice per genotype, sex, and age used in behavioral and molecular analyses.

Genotype	Sex	Total	2 mo	4 mo	6 mo	8 mo	16 mo	Additional Samples Used for Non-Behavioral Studies (Group Age)
WT	Male	37	6	9	9	6	5	2 (16 mo)
MFS	Male	31	6	7	7	4	4	3 (16 mo)
WT	Female	34	6	8	8	5	4	3 (16 mo)
MFS	Female	32	6	7	7	4	5	1 (8 mo) + 2 (16 mo)
Total	—	134	24	31	31	19	18	11

Mo, months old.

**Table 2:** Primer sequences used for real-time quantitative PCR studies.

Gene	RefSeq mRNA Accession Number	Forward (5′-3′)	Reverse (5′-3′)
*Gria1*	NM_008165.4	GAGCAACGAAAGCCCTGTGA	CCCTTGGGTGTCGCAATG
*Gria2*	NM_013540.4	AAAGAATACCCTGGAGCACAC	CCAAACAATCTCCTGCATTTCC
*Grin1*	NM_001372559.1	CCGTGAACGTGTGGAGGAA	TCTGCTCTACCACTCTTTCTATCCTG
*Hmox1*	NM_010442.2	CACGCATATACCCGCTACCT	CCAGAGTGTTCATTCGAGCA
*Il6*	NM_031168.2	AACCACGGCCTTCCCTACTTCA	TCATTTCCACGATTTCCCAGAG
*Nos2*	NM_010927.4	GGCCAGCCTGTGAGACCTTT	TTGGAAGTGAAGCGTTTCG
*Nox1*	NM_172203.2	CCCAGCAGAAGGTCGTGATT	GCTAAAGCCTCGCTTCCTCAT
*Nox2*	NM_007807.5	CAGGAACCTCACTTTCCATAAGAT	AACGTTGAAGAGATGTGCAATTGT
*Nox4*	NM_015760.5	CCGGACAGTCCTGGCTTATCT	TGCTTTTATCCAACAATCTTCTTGTT
*Tgfb1*	NM_011577.2	CCGCAACAACGCCATCTATG	CCCGAATGTCTGACGTATTGAAG
*Tnf*	NM_013693.2	AGGCACTCCCCCAAAAGATG	TCACCCCGAAGTTCAGTAGAC
*Trpv1*	NM_001001445.2	CATGCTCATTGCTCTCATGG	TCCTCATGCACTTCAGGAAA
*Oprm1*	NM_011013	TCCTGGTCATGTATGTGATTGTAAGA	CGTGCTAGTGGCTAAGGCATCT

*Gria1*/*Gria2*, AMPA (α-amino-3-hydroxy-5-methyl-4-isoxazolepropionic acid) receptor subunits; *Grin1*, NMDA (N-methyl-D-aspartate) receptor subunit; *Hmox1*, heme oxygenase-1; *Il6*, interleukin-6; *Nos2*, inducible nitric oxide synthase; *Nox*, nicotinamide adenine dinucleotide phosphate oxidase; *Tgfb1*, transforming growth factor beta 1; *Tnf*, tumor necrosis factor α; *Trpv1*, transient receptor potential vanilloid 1; *Oprm1*, μ-opioid receptor.

## Data Availability

Data is contained within the article.

## Supplementary Materials

The following supporting information can be downloaded at https://www.mdpi.com/article/10.3390/antiox15010080/s1. Figure S1: Heat map depicting the log-normalized expression levels of genes involved in opioid and heme oxygenase 1 (HMOX1) signaling. Figure S2: Development of increased large elastin breaks in MFS mice. Figure S3: Expression of *Tgfb1*, *Hmox1*, and *Oprm1* in the spinal cord of MFS mice at 2 and 16 months of age. Figure S4: Levels of some representative redox markers in the plantar skin of MFS mice at 16 months of age. Table S1: Top differentially expressed genes in brain tissue from Marfan syndrome (MFS) vs. wild-type (WT) mice. Genes are grouped by sex and age. Accession numbers correspond to Ensembl Mouse Gene IDs.
