# Supplementary material for: Pain Hypersensitivity in a Mouse Model of Marfan Syndrome
Source: Antioxidants (Basel). 2026 Jan 8;15(1):80. doi: 10.3390/antiox15010080 (PMC12837253; doi:10.3390/antiox15010080)
Supplement: Supplementary file 1 [file antioxidants-15-00080-s001.zip › Supplementary Figure S1.pdf]

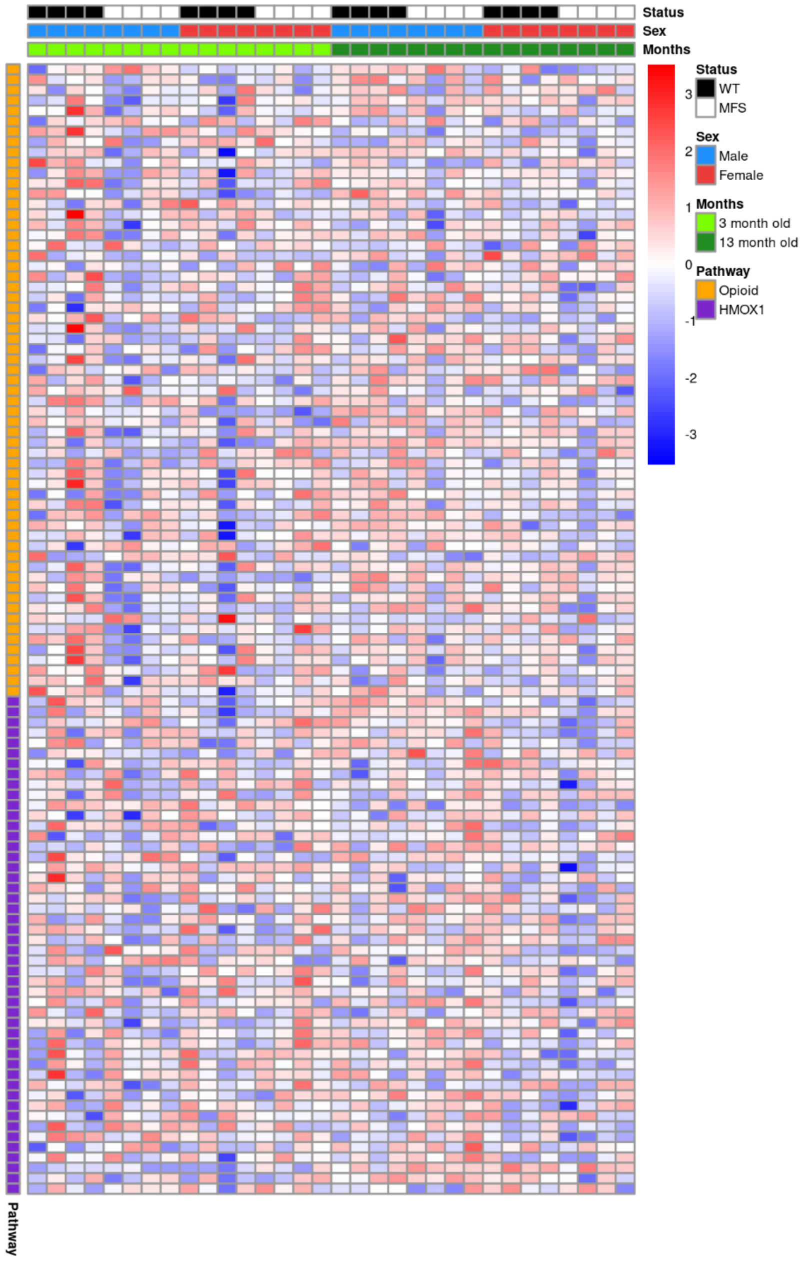

Supplementary Figure S1. Heat map depicting the log-normalized expression levels of genes involved in opioid and heme oxygenase 1 (HMOX1) signaling. REACTOME pathways in the brains of 3- and 13-month-old male and female wild-type (WT) and Marfan syndrome (MFS) mice. Lower and higher expression levels are represented in blue and red, respectively. Each row corresponds to a single gene, and each column represents a sample. N = 4 per experimental group.
