# Supplementary material for: Pain Hypersensitivity in a Mouse Model of Marfan Syndrome
Source: Antioxidants (Basel). 2026 Jan 8;15(1):80. doi: 10.3390/antiox15010080 (PMC12837253; doi:10.3390/antiox15010080)
Supplement: Supplementary file 1 [file antioxidants-15-00080-s001.zip › Supplementary Figure S2.pdf]

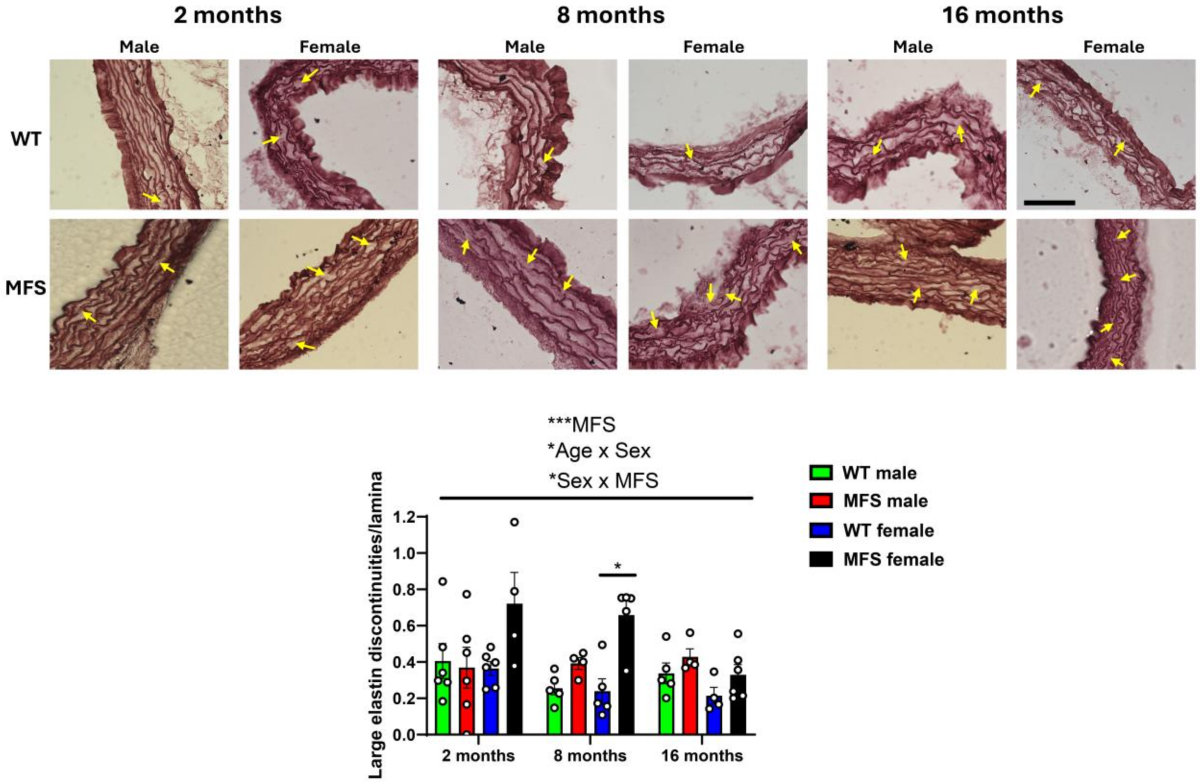


**Supplementary Figure S2. Development of increased large elastin breaks in MFS mice.** Representative images (top) and quantification (bottom) of large (≥ 20 μm) elastin discontinuities per lamina in Orcein-stained histological sections of the ascending aorta from 2-, 8-, and 16-month-old WT and MFS male and female mice. Scale bar = 100 μm. Representative large elastin breaks are indicated by yellow arrows. Data are expressed as mean values ± SEM. *$p < 0.05$ and ***$p < 0.001$ by three-way ANOVA with Sidak test.
