# Supplementary material for: Pain Hypersensitivity in a Mouse Model of Marfan Syndrome
Source: Antioxidants (Basel). 2026 Jan 8;15(1):80. doi: 10.3390/antiox15010080 (PMC12837253; doi:10.3390/antiox15010080)
Supplement: Supplementary file 1 [file antioxidants-15-00080-s001.zip › Supplementary Figure S3.pdf]

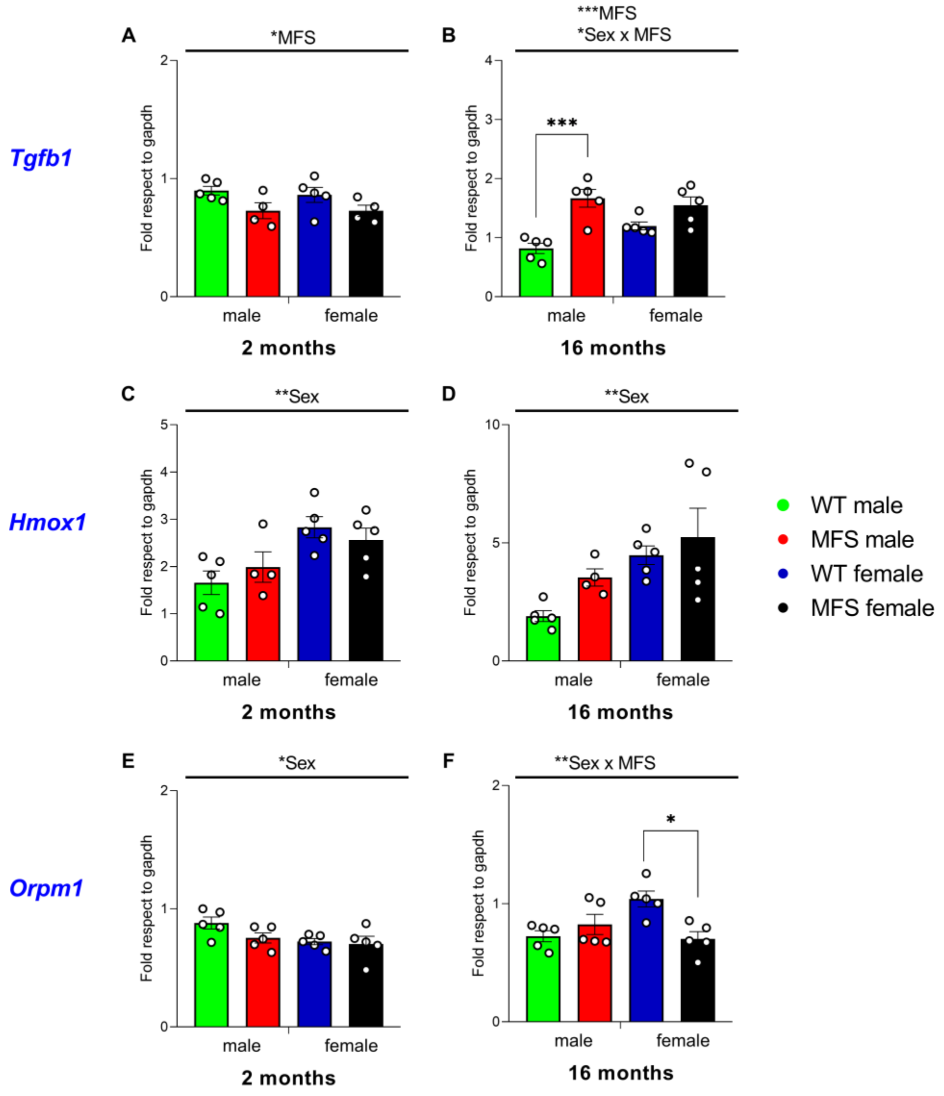

Supplementary Figure S3. Expression of *Tgfb1*, *Hmox1*, and *Oprm1* in the spinal cord of MFS mice at 2 and 16 months of age. Data are presented as fold respect to GAPDH for *Tgfb1* (A,B), *Hmox1* (C,D), and *Oprm1* (E,F) in the spinal cord of male and female WT and MFS animals at 2 and 16 months of age. In all panels, symbols show the level of statistical significance; * $p < 0.05$, ** $p < 0.01$, and *** $p < 0.001$ (one-way ANOVA followed by Sidak test). Data are expressed as mean values ± SEM; n = 4-5 samples per group.
