# Supplementary material for: Pain Hypersensitivity in a Mouse Model of Marfan Syndrome
Source: Antioxidants (Basel). 2026 Jan 8;15(1):80. doi: 10.3390/antiox15010080 (PMC12837253; doi:10.3390/antiox15010080)
Supplement: Supplementary file 1 [file antioxidants-15-00080-s001.zip › Supplementary Figure S4.pdf]

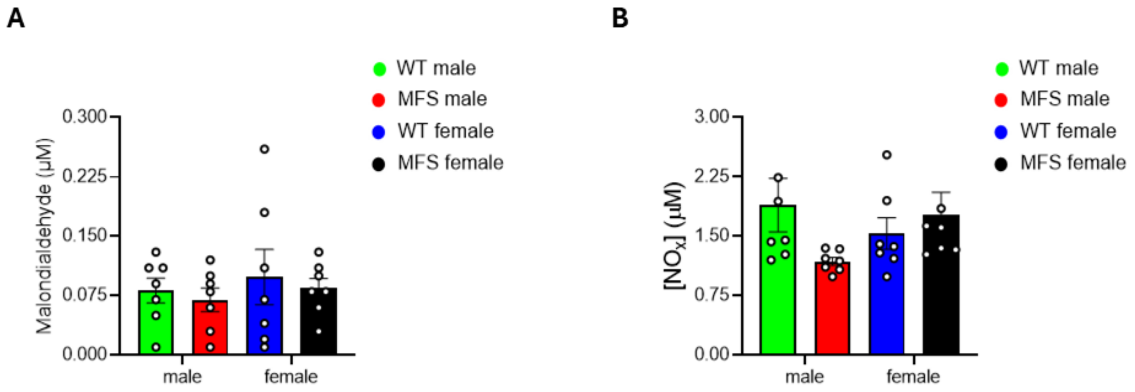

Supplementary Figure S4. Levels of some representative redox markers in the plantar skin of MFS mice at 16 months of age. (A) Malondialdehyde concentrations, as an indirect indicator of lipid oxidation, measured by spectrophotometry. (B) Nitric oxide metabolites (nitrites and nitrates; NO_x) concentrations measured by spectrophotometry. Data are expressed as mean values ± SEM.
