# Supplementary material for: Pain Hypersensitivity in a Mouse Model of Marfan Syndrome
Source: Antioxidants (Basel). 2026 Jan 8;15(1):80. doi: 10.3390/antiox15010080 (PMC12837253; doi:10.3390/antiox15010080)
Supplement: Supplementary file 1 [file antioxidants-15-00080-s001.zip › Supplementary Table S1.pdf]

Supplementary Table S1. Top differentially expressed genes in brain tissue from Marfan syndrome (MFS) vs. wild-type (WT) mice. Genes are grouped by sex and age. Accession numbers correspond to Ensembl Mouse Gene IDs.

Gene symbol	Accession number	Regulation	log2FC	*p* value	Adjusted *p* value
**Male, 3-month-old mice (MFS vs. WT)**					
Kcnip3	ENSMUSG00000079056.13	Down	-0.296	1.69e-05	0.271
Myl9	ENSMUSG00000067818.7	Up	0.367	2.47e-04	0.517
Fahd2a	ENSMUSG00000027371.11	Up	0.303	3.26e-04	0.517
Gm10432	ENSMUSG00000113679.2	Up	0.436	4.58e-04	0.517
Fjx1	ENSMUSG00000075012.5	Down	-0.301	6.04e-04	0.517
Ldb3	ENSMUSG00000021798.15	Up	0.297	6.41e-04	0.517
AW011738	ENSMUSG00000078349.5	Up	0.362	6.67e-04	0.517
Avpi1	ENSMUSG00000018821.4	Down	-0.314	6.86e-04	0.517
C130074G19Rik	ENSMUSG00000039349.6	Down	-0.433	8.02e-04	0.517
Dzip1	ENSMUSG00000042156.17	Up	0.220	8.05e-04	0.517
**Male, 13-month-old mice (MFS vs. WT)**					
Kcnip3	ENSMUSG00000079056.13	Down	-0.304	1.21e-05	0.193
Fahd2a	ENSMUSG00000027371.11	Up	0.330	8.18e-05	0.656
C4b	ENSMUSG00000073418.5	Up	0.310	6.25e-04	1.000

Kash5	ENSMUSG00000038292.15	Down	-0.452	8.08e-04	1.000
Pfn4	ENSMUSG00000120639.1	Down	-0.531	8.45e-04	1.000
Cybc1	ENSMUSG00000039294.15	Up	0.196	9.87e-04	1.000
Fgfbp3	ENSMUSG00000047632.12	Down	-0.403	1.10e-03	1.000
Fbxo38	ENSMUSG00000042211.8	Up	0.121	1.24e-03	1.000
Tex52	ENSMUSG00000079304.6	Up	0.373	1.52e-03	1.000
Sspn	ENSMUSG00000030255.14	Up	0.239	1.92e-03	1.000
**Female, 3-month-old mice (MFS vs. WT)**					
Pcdhga8	ENSMUSG00000103897.2	Up	0.441	4.16e-07	0.007
Kcnip3	ENSMUSG00000079056.13	Down	-0.274	4.95e-05	0.262
9430091E24Rik	ENSMUSG00000084808.8	Down	-0.800	5.69e-05	0.262
Arap3	ENSMUSG00000024451.10	Down	-0.295	7.38e-05	0.262
Pcdhga9	ENSMUSG00000102440.2	Up	0.371	8.17e-05	0.262
Cdk14	ENSMUSG00000028926.16	Up	0.174	2.92e-04	0.777
Zc3hav1	ENSMUSG00000029826.15	Up	0.446	3.47e-04	0.777
Pcdhga10	ENSMUSG00000102222.2	Up	0.264	4.41e-04	0.777
Sag	ENSMUSG00000056055.14	Down	-0.630	5.54e-04	0.777
Mrrf	ENSMUSG00000026887.10	Up	0.183	6.51e-04	0.777
**Female, 13-month-old mice (MFS vs. WT)**					

Gm5537	ENSMUSG00 000069008.4	Down	-1.091	1.30e-04	1.000
Cxcl12	ENSMUSG00 000061353.12	Up	0.302	6.34e-04	1.000
Smg8	ENSMUSG00 000020495.14	Down	-0.189	6.46e-04	1.000
Gm26920	ENSMUSG00 000058447.9	Down	-5.068	8.37e-04	1.000
Sh3bp1	ENSMUSG00 000022436.17	Up	0.280	1.06e-03	1.000
Fahd2a	ENSMUSG00 000027371.11	Up	0.267	1.06e-03	1.000
Gm7061	ENSMUSG00 000082776.4	Up	0.858	1.17e-03	1.000
Evc	ENSMUSG00 000029122.12	Up	0.279	1.21e-03	1.000
Tyrobp	ENSMUSG00 000030579.11	Down	-0.266	1.60e-03	1.000
Gm49358	ENSMUSG00 000112743.2	Down	-4.438	1.66e-03	1.000

Genes annotated with “*Gm*” or “*Rik*” represent predicted mouse loci or transcripts with limited functional characterization.
